# Laparoscopic right hemicolectomy for cystic lymphangioma of the right mesocolon: a case report

**DOI:** 10.1093/jscr/rjaf832

**Published:** 2025-10-17

**Authors:** Akira Yamamoto, Rieko Takagi, Mayu Yamamoto, Honoka Yokoyama, Yuki Morimoto, Takeshi Yokoe, Eiki Ojima, Keiichi Uchida, Yasuhiko Mohri

**Affiliations:** Department of Gastrointestinal and General Surgery, Mie Prefectural General Medical Center, 5450-132, Hinaga, Yokkaichi, Mie 514-8561, Japan; Department of Gastrointestinal and Pediatric Surgery, Division of Reparative Medicine, Institute of Life Sciences, Mie University Graduate School of Medicine, 2-174, Edobashi, Tsu, Mie 514-8507, Japan; Department of Gastrointestinal and General Surgery, Mie Prefectural General Medical Center, 5450-132, Hinaga, Yokkaichi, Mie 514-8561, Japan; Department of Gastrointestinal and General Surgery, Mie Prefectural General Medical Center, 5450-132, Hinaga, Yokkaichi, Mie 514-8561, Japan; Department of Gastrointestinal and General Surgery, Mie Prefectural General Medical Center, 5450-132, Hinaga, Yokkaichi, Mie 514-8561, Japan; Department of Gastrointestinal and General Surgery, Mie Prefectural General Medical Center, 5450-132, Hinaga, Yokkaichi, Mie 514-8561, Japan; Department of Gastrointestinal and General Surgery, Mie Prefectural General Medical Center, 5450-132, Hinaga, Yokkaichi, Mie 514-8561, Japan; Department of Gastrointestinal and General Surgery, Mie Prefectural General Medical Center, 5450-132, Hinaga, Yokkaichi, Mie 514-8561, Japan; Department of Pediatric Surgery, Mie Prefectural General Medical Center, 5450-132 Hinaga, Yokkaichi, Mie 514-8561, Japan; Department of Gastrointestinal and General Surgery, Mie Prefectural General Medical Center, 5450-132, Hinaga, Yokkaichi, Mie 514-8561, Japan

**Keywords:** mesocolic lymphangioma, laparoscopic surgery, mesenteric cyst, right hemicolectomy

## Abstract

A 67-year-old man presented with an abdominal mass and was diagnosed with a mesenteric cyst based on imaging studies. The cyst was extensively located within the mesentery of the ascending colon; however, complete resection was deemed feasible by dissecting along the right margin of the superior mesenteric vein. A laparoscopic right hemicolectomy was planned and successfully performed, despite the technical challenge posed by adhesive fascial scarring. Thorough preoperative evaluation allowed for safe and complete resection using a minimally invasive approach.

## Introduction

Lymphangiomas are relatively rare benign tumors considered to result from malformations of the lymphatic drainage system, and they predominantly present in childhood [1]. These cystic tumors most commonly occur in the head, neck, and axillary regions of pediatric patients, where they are often asymptomatic and discovered incidentally [2]. Occurrence in the mediastinum, retroperitoneum, or abdominal cavity—where they may appear as mesenteric cysts—is uncommon. In adults, intra-abdominal lymphangiomas of unknown origin are particularly rare, accounting for ~1 in 100 000 hospital admissions [3]. The condition is reported to be more frequent in males, with a male-to-female ratio ranging from 1.5:1 to 3:1. To date, fewer than 200 cases of mesenteric lymphangiomas have been reported in the literature. Here, we report a rare case of a right mesocolic lymphangioma that was successfully resected laparoscopically.

## Case presentation

A 67-year-old man was referred to our institution with a primary complaint of an abdominal mass, without associated gastrointestinal symptoms such as abdominal pain or diarrhea. On physical examination, a non-tender palpable mass was detected in the right upper quadrant.

Laboratory investigations, including complete blood count and biochemical analyses, were within normal limits. Although the soluble interleukin-2 receptor level was elevated, tumor markers such as carcinoembryonic antigen (CEA), carbohydrate antigen 19–9 (CA 19–9), and squamous cell carcinoma antigen (SCC) were within normal ranges. Colonoscopy and barium enema revealed colonic diverticula but showed no evidence of obstruction or malignancy.

**Figure 1:**
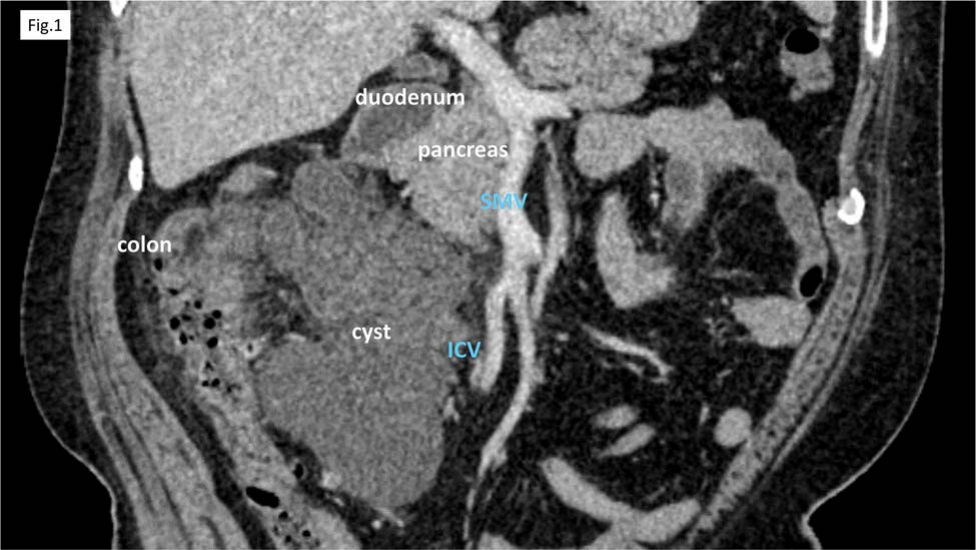
CT showed cystic lesions localized within the right colic mesentery.

**Figure 2:**
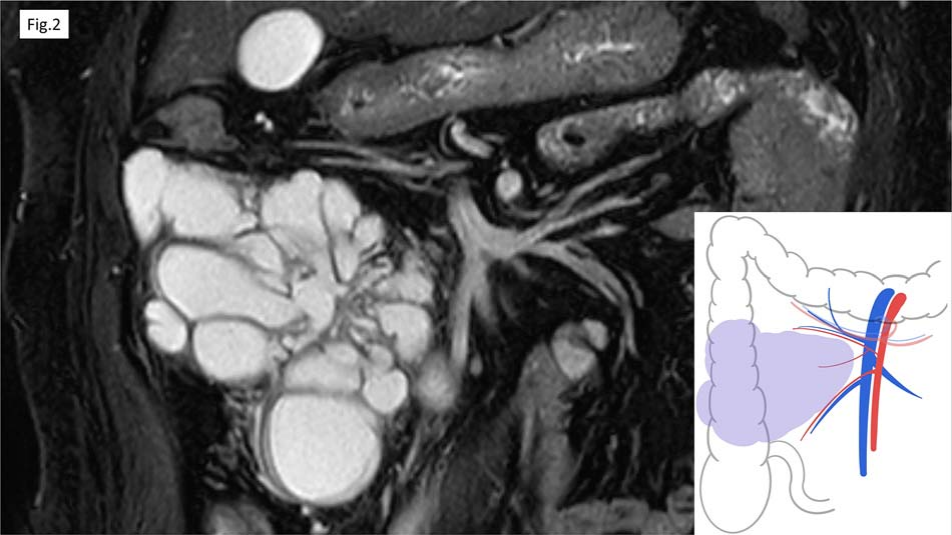
MRI revealed the multifocal cystic lesions, which lacked distinct enhancement.

Computed tomography (CT) revealed cystic lesions located in the mesentery of the right colon, without apparent connection to the gastrointestinal tract (Fig. 1). Magnetic resonance imaging (MRI) confirmed the presence of multiple non-enhancing cystic lesions (Fig. 2). Radiological findings suggested that the cysts were confined to the mesentery of the ascending colon, delineated by the ileocolic artery and vein (ICA/V), the right margin of the superior mesenteric vein (SMV), and the right branch of the middle colic artery (MCA-rt) and accessory right colic vein (aRCV).

**Figure 3:**
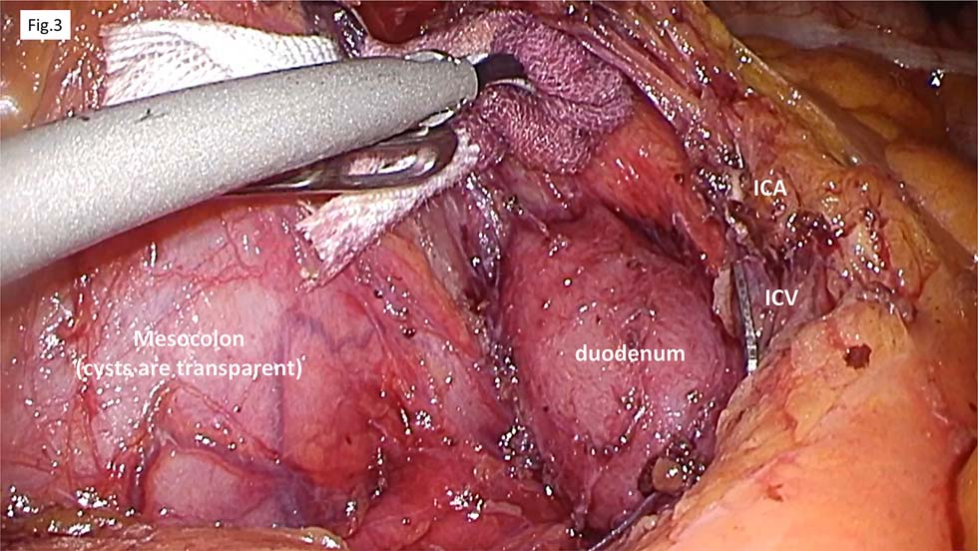
Operative findings. The procedure began with a mesenteric incision at the caudal level of the ICA/V. Dissection along the fusion fascia confirmed the preservation of the duodenum. We addressed the mesentery containing the cyst by ligating the ICA/V, making an incision along the right margin of the SMV, and resected the accessory right colic vein.

A laparoscopic right hemicolectomy was performed. The mesentery was incised caudal to the ICA, and dissection proceeded along the fusion fascia, allowing preservation of the duodenum. In accordance with the preoperative plan, the involved mesentery was resected by ligating the ICA/V, incising along the right margin of the SMV, and dividing the aRCV (Fig. 3). Although dissection was challenging due to fibrotic, scar-like changes in the fascia—likely a result of chronic inflammation surrounding the cyst—the procedure was completed as planned, including mobilization of the hepatic flexure and lateral attachments. Considering potential impairment of blood supply from mesenteric resection, bowel transection and functional end-to-end anastomosis were performed.

Postoperatively, gastric decompression was continued until postoperative day (POD) 2 due to concern about duodenal handling. Oral intake was initiated on POD 5, and the patient was discharged without complications on POD 9.

**Figure 4:**
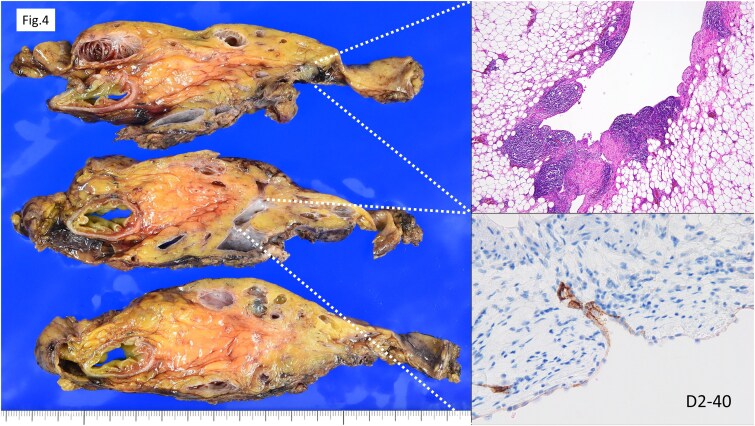
Histopathological findings. Cysts of varying sizes were observed within the mesentery. The cyst lumens exhibited smooth contours with no evidence of enhancing lesions. The cyst linings consisted of a sparse single layer of flat to cuboidal cells. D2–40 showed partial positivity but with inconsistent results. These findings suggested the possibility of lymphangiomatosis.

Histopathological examination (Fig. 4) revealed multiple cysts of varying sizes within the mesentery. The cyst lumens were smooth and lacked solid or enhancing components. The lining consisted of a sparse single layer of flat to cuboidal cells. The cyst walls contained mature smooth muscle with mild lymphocytic infiltration and lymphoid follicle formation. There was minimal nuclear atypia, and no malignant features were observed. Immunohistochemically, the cyst-lining cells were negative for calretinin and CD34. D2–40 staining showed focal but inconsistent positivity (Supplementary Fig. 1). These findings were suggestive of mesenteric lymphangiomatosis.

## Discussion

Mesenteric lymphatic cysts were first formally described by Rokitansky [4]. Lymphangiomas are believed to result from impaired lymphatic drainage, either due to congenital malformations or acquired factors. These lesions typically present as multifocal cysts filled with lymphatic fluid. Histologically, lymphangiomas are classified into three main types: capillary, cavernous, and cystic. While capillary and cavernous types often present as cutaneous lesions, the cystic type commonly occurs within the abdominal or retroperitoneal cavities [5].

Several theories have been proposed regarding their etiology. One suggests that cystic lymphangiomas arise from the proliferation and dilation of blind-ended lymphatic sacs that fail to connect with the venous system during embryologic development [6]. Other proposed causes include developmental anomalies, trauma, previous abdominal surgeries, radiation exposure, lymphatic obstruction, or chronic inflammation. However, these theories remain speculative, as neither intraoperative findings nor pathological analyses in most cases—including ours—have confirmed such mechanisms [7].

Due to the rarity of colonic lymphangiomas, there is no consensus regarding optimal management. Treatment strategies are typically guided by factors such as the lesion’s location, size, growth rate, and presence of symptoms [8]. These lesions may grow progressively and may involve or compress adjacent structures, potentially leading to complications [8]. While malignant transformation is not commonly associated with lymphangiomas, malignancy should always be considered in the differential diagnosis, particularly when imaging findings are atypical or the lesion is large [9].

In our case, the patient presented with an abdominal mass, and although we could not determine the growth rate, imaging showed multiple cysts occupying the right mesocolon. Given the size, location, and potential for further expansion, surgical resection was considered appropriate.

Complete surgical resection is the most commonly recommended treatment. Recurrence, when reported, is usually attributed to incomplete resection or regrowth of residual tissue, rather than true recurrence or metastasis as seen in malignant tumors [10]. In this case, preoperative imaging—CT and MRI—suggested that complete resection would be possible by applying the principles of complete mesocolic excision. When the relationship between the cystic lesion and surrounding vessels can be clearly visualized preoperatively, laparoscopic resection is feasible. Care must be taken to avoid rupture or incomplete resection, especially since other differential diagnoses—such as cystic mucinous adenoma or cystic mesothelioma—may present similarly. An additional intraoperative consideration is the fusion fascia, which may appear fibrotic or scar-like, as observed in our case, thereby complicating dissection.

In conclusion, although mesocolic lymphangiomas are rare and resection cases are infrequently reported, our experience demonstrates that complete laparoscopic excision is feasible when preoperative imaging enables a clear understanding of vascular anatomy and lesion boundaries.

## Supplementary Material

RSI_rjaf832

## Data Availability

Data sharing is not applicable to this article because no datasets were generated or analyzed.

## References

[1] Kambakamba P, Lesurtel M, Breitenstein S, et al. Giant mesenteric cystic lymphangioma of mesocolic origin in an asymptomatic adult patient. J Surg Case Rep 2012;2012:4. 10.1093/jscr/2012.6.4 PMC3862254 24960673

[2] Jeong WK, Kim Y, Song SY, et al. Cavernous mesenteric lymphangioma (2006, 4b). Eur Radiol 2006;16:1625–8. 10.1007/s00330-005-0106-0 16770656

[3] Kinas V . Mesenteric cystic lymphangioma: a case report. J Kartal Training Res Hospital 2016;27:258–60.

[4] Kurtz RJ, Heimann TM, Holt J, et al. Mesenteric and retroperitoneal cysts. Ann Surg 1986;203:109–12. 10.1097/00000658-198601000-00017 3942415 PMC1251046

[5] Losanoff JE, Richman BW, El-Sherif A, et al. Mesenteric cystic lymphangioma. J Am Coll Surg 2003;196:598–603. 10.1016/S1072-7515(02)01755-6 12691938

[6] Cohen SR, Thompson JW. Lymphangiomas of the larynx in infants and children. A survey of pediatric lymphangioma. Ann Otol Rhinol Laryngol Suppl 1986;127:1–20. 3098156 10.1177/00034894860950s601

[7] Mir AS, Abel WF, Lebel DP. Colonic lymphangiomatosis. Cureus 2023;15:e39085. 10.7759/cureus.39085 37332407 PMC10269742

[8] Zhuo CH, Shi DB, Ying MG, et al. Laparoscopic segmental colectomy for colonic lymphangiomas: a definitive, minimally invasive surgical option. World J Gastroenterol 2014;20:8745–50. 10.3748/wjg.v20.i26.8745 25024636 PMC4093731

[9] Pham HD, Nguyen TA, Doan TG, et al. Lymphangioma of colon presenting as an intramural tumor. Int Med Case Rep J 2022;15:361–6. 10.2147/IMCRJ.S368610 35845223 PMC9285534

[10] Walker AR, Putnam TC. Omental, mesenteric, and retroperitoneal cysts: a clinical study of 33 new cases. Ann Surg 1973;178:13–9. 10.1097/00000658-197307000-00003 4717705 PMC1355855

