# Supplementary figures and images for: Laparoscopic right hemicolectomy for cystic lymphangioma of the right mesocolon: a case report
Source: J Surg Case Rep. 2025 Oct 17;2025(10):rjaf832. doi: 10.1093/jscr/rjaf832 (PMC12531991; doi:10.1093/jscr/rjaf832)

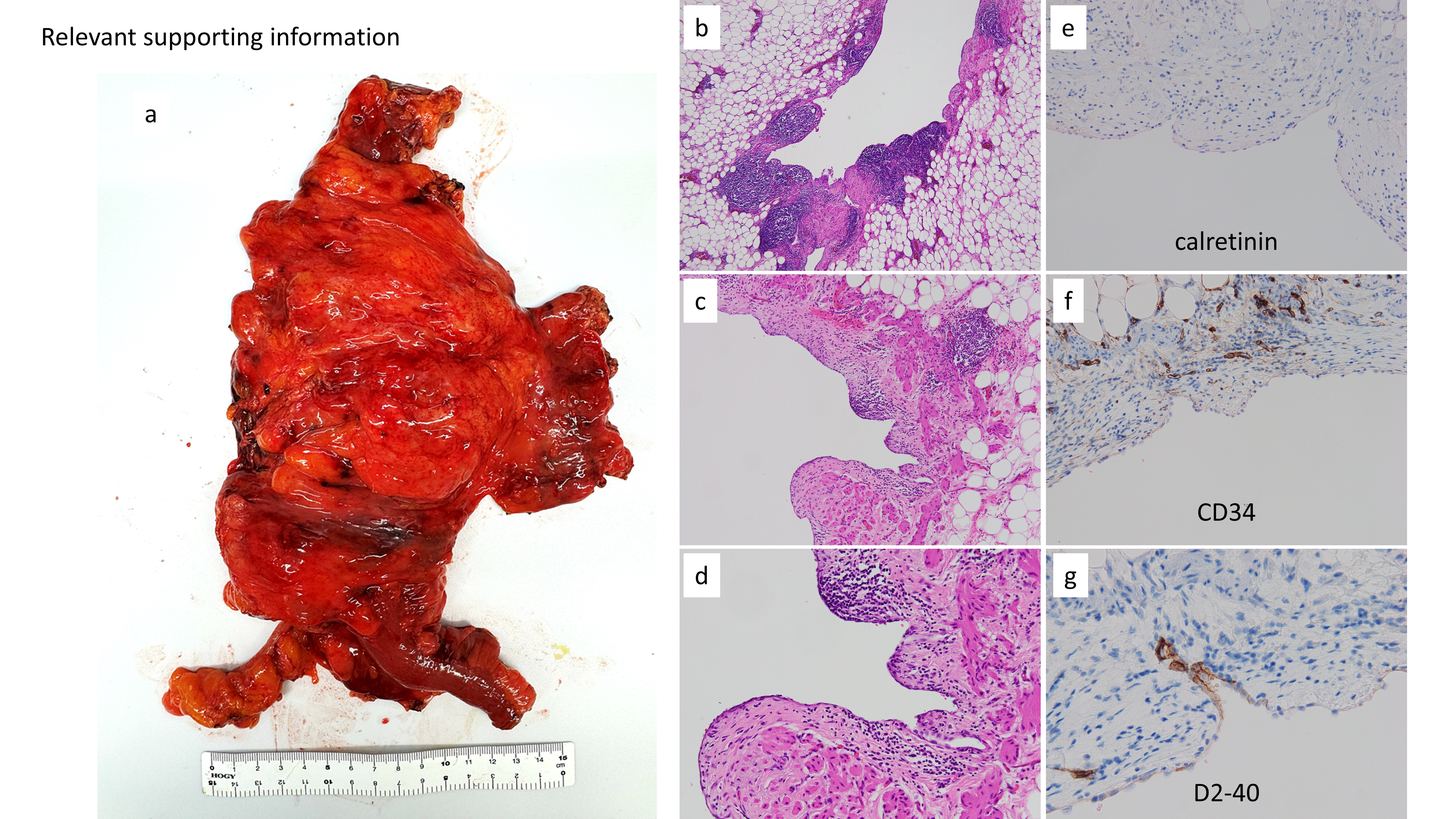

Supplement: RSI_rjaf832 [file rsi_rjaf832.jpeg]
